# Clinical Outcomes and Disease Burden in Amyloidosis Patients with and Without Atrial Fibrillation—Insight from the National Inpatient Sample Database

**DOI:** 10.19102/icrm.2021.120605

**Published:** 2021-06-15

**Authors:** Shakeel Jamal, Asim Kichloo, Beth Bailey, Jagmeet Singh, Hafeez Virk, Ronak Soni, Farah Wani, Muhammad Ajmal, Sindhura Ananthaneni, Ehizogie Edigin, Rajeev Sudhakar, Khalil Kanjwal

**Affiliations:** ^1^Department of Internal Medicine, Central Michigan University, College of Medicine, Saginaw, MI, USA; ^2^Division of Nephrology, Geisinger Commonwealth School of Medicine, Scranton, PA, USA; ^3^Division of Cardiovascular Medicine, Albert Einstein College of Medicine, Philadelphia, PA, USA; ^4^Division of Cardiovascular Medicine, University of Toledo, College of Medicine, Toledo, OH, USA; ^5^Department of Internal Medicine, Samaritan Medical Center, Watertown, NY, USA; ^6^Division of Cardiovascular Medicine, University of Arizona, College of Medicine, Tucson, AZ, USA; ^7^Department of Internal Medicine, John H Stroger Hospital of Cook County, Chicago, IL, USA; ^8^Division of Cardiovascular Medicine, Ascension Medical Group, Central Michigan University, Saginaw, MI, USA; ^9^Division of Electrophysiology, McLaren Greater Lansing Hospital, Michigan State University, Lansing, MI, USA

**Keywords:** Amyloidosis, atrial fibrillation, disease burden, National Inpatient Sample

## Abstract

Amyloidosis is a systemic illness that affects multiple organ systems, including the cardiovascular, renal, gastrointestinal, and pulmonary systems. Common manifestations include restrictive cardiomyopathy, arrhythmias, nephrotic syndrome, and gastrointestinal hemorrhage. It is unknown whether coexisting atrial fibrillation (AF) worsens the disease burden and outcomes in patients with systemic amyloidosis. In this study, those with a diagnosis of amyloidosis with and without coexisting AF were identified by querying the Healthcare Cost and Utilization Project—specifically, the National Inpatient Sample for the year 2016—based on International Classification of Diseases, 10th Revision, Clinical Modification codes. During 2016, a total of 2,997 patients were admitted with a diagnosis of amyloidosis, including 918 with concurrent AF. Greater rates of mortality (7.4% vs. 5.6%); heart block (6.8% vs. 2.8%); cardiogenic shock (5% vs. 1.6%); placement of an implantable cardioverter-defibrillator, cardiac resynchronization therapy device, or permanent pacemaker (14.5% vs. 4.5%); renal failure (29% vs. 21%); heart failure (66% vs. 30%); and bleeding complications (5.7% vs. 2.8%) were observed in patients with a diagnosis of amyloidosis and coexisting AF when compared with in patients without AF. Interestingly, patients with amyloidosis without comorbid AF had greater odds of associated stroke relative to those with concurrent AF (7.9% vs. 3.4%).

## Introduction

Amyloidosis is a heterogeneous group of inherited or acquired diseases that results from extracellular systemic deposition of amyloid, which involves 20 different insoluble fibrillary proteins assembled in an abnormal β-sheet conformation.^1,2^ Amyloidosis can result in the deposition of these proteins in various organ systems, including the heart, kidneys, central nervous system, blood vessels, pancreas, and liver, disrupting organ integrity, structure, and function. This process can lead to multiple organ system involvement and can manifest either in the form of a serious illness or just an incidental finding.^3^ Treatment strategies are aimed at destabilizing these fibrillary deposits.^4^ Systemic amyloidosis can manifest in the form of amyloid A (AA) amyloidosis and amyloid light-chain (AL) amyloidosis (most common). AL amyloidosis results from the accumulation of fibrils derived from monoclonal immunoglobulin light chains, which can involve any organ system in the body directly, excluding the brain, and the most common causes of death in patients with the condition are heart failure, uremia, and autonomic neuropathy.^5,6^ AA amyloidosis is also known as reactive amyloidosis as it is a consequence of persistent inflammatory, neoplastic, and infective disorders. Hereditary systemic amyloidosis is an autosomal dominant condition in which amyloid fibrils are formed from genetic variants of apolipoproteins, transthyretin, lysozyme, and fibrinogen A-α.^7^

Amyloidosis is known to affect any organ system in the body; the purpose of this study was to understand the outcomes on the cardiovascular system in general and in concert with atrial fibrillation (AF) specifically. Cardiac involvement is thought to be the leading cause of morbidity and mortality in amyloidosis.^8^ Fifty percent of patients with AL amyloidosis have an underlying cardiac involvement, which depends on the type of amyloid.^9^ Amyloid deposits cause myocardial cell toxicity by the deposition of amyloid fibrils in the extracellular space of cardiac myocytes in the atria, the ventricles, around the coronaries, and in the valves,^10^ which leads to restrictive cardiomyopathy, continuing further to diastolic dysfunction and manifesting during physical examinations as edema, congestive hepatomegaly, and jugular venous distension, while patients with progression to advanced forms of the disease present with hypotension.^11^ Atrial arrhythmias occur most commonly in up to 10% to 15% of patients. AF is known to be the most common atrial arrhythmia in amyloidosis.^8^ Deposition of amyloid in the atrial issue leads to altered conduction, which increases the likelihood of triggering AF, while AF increases the likelihood of the deposition of amyloid, creating a vicious cycle.^12^ Sanchis et al. reported 238 cases of cardiac amyloidosis in a study where 48% had AL amyloidosis and 52% had transthyretin amyloid (ATTR) cardiomyopathy. Forty-two percent of the 238 study participants had AF, 60% had permanent AF, and 40% had nonpermanent AF.^13^ Another study looked at 382 patients with ATTR cardiomyopathy, in which 69% were found to have AF (stratified as 45% with paroxysmal, 27% with persistent, 15% with longstanding persistent, and 13% with permanent AF).^14^ Krishnappa et al. studied the cardiac biopsies of 1,083 patients with AF and found that 3.1% had subclinical cardiac amyloidosis.^15^ Röcken et al. observed an association between isolated atrial amyloidosis and AF irrespective of age and sex based on biopsies of the right atrial appendage collected during open-heart surgeries.^12^ AL amyloidosis can have a spectrum ranging from no involvement of the heart at all to a severe form of cardiovascular disease.^16^ Cardiac amyloidosis presents with restrictive cardiomyopathic symptoms (exercise intolerance) followed by systolic dysfunction, atrial/ventricular arrhythmias [amyloid deposits in the sinoatrial/atrioventricular (AV) node and conduction tissue], myocardial infarction (deposition of amyloid deposits in coronaries), and outflow tract obstruction due to asymmetric deposition of amyloid fibrils in the interventricular septum.^8,17–19^ Pericardial and pleural effusion have also been reported.^20,21^

We queried the National Inpatient Sample (NIS) to investigate the hypothesis that coexisting AF worsens the clinical outcomes and associated disease burden in patients with amyloidosis and that appropriate management of AF may improve the clinical outcomes and disease burden. To evaluate our hypothesis, we conducted a cross-sectional analysis.

## Methods

### Data source

The NIS has been elaborated on in detail in prior studies.^22^ The NIS is the largest publicly available database in the United States (US), which falls under the Healthcare Cost and Utilization Project (HCUP) and is maintained by the Agency for Healthcare Research and Quality. It is one of the most useful databases available to study the outcomes and trends of various procedures and diseases. It includes deidentified data collected from 20% of community hospitals of 46 states in the US. Each hospitalization is representative of one primary diagnosis, up to 29 secondary diagnoses, and 15 procedures using the international Clinical Modification codes [International Classification of Diseases, ninth revision and 10th revision (ICD-10)]. The available data include admission status, demographics, admitting diagnosis, comorbidities, health care facility (whether rural or urban), discharge diagnosis, outcomes, length of stay, and costs during hospitalization. It is important to mention here the inherent limitations of the NIS, including a lack of distinction between acute and chronic diagnoses and between comorbidities and complications; however, we evaluated the disease burden and inpatient mortality and length of stay as outcomes.^23,24^

We examined all adult patients who were hospitalized in the US during the year 2016 with the diagnosis of amyloidosis and with comorbid AF using the NIS. Patients were filtered using ICD-10 Clinical Modification codes. We identified all adult patients aged 18 years or more who were admitted with amyloidosis with and without a concomitant primary or secondary diagnosis with AF during the year 2016. We excluded any hospitalizations with missing demographics (ie, age, sex, admission or discharge diagnosis, and mortality data). We utilized NIS variables to identify patients’ age, sex, race, county location, county income, hospital bed size, and alcohol abuse. Race was divided into two categories, white and nonwhite.

### Primary outcomes and comorbid conditions

Our objectives were to assess the comorbidities and inpatient outcomes in patients with a diagnosis of amyloidosis with and without coexisting AF. The primary outcomes analyzed were inpatient mortality and length of the hospital stay of all patients admitted with amyloidosis with and without coexisting AF. Comorbidities associated with amyloidosis and AF were stroke; heart failure; bleeding; supraventricular tachycardia; ventricular tachycardia; heart block; cardiac arrest; cardiogenic shock; implantable cardioverter-defibrillator (ICD), permanent pacemaker (PPM), or cardiac resynchronization therapy (CRT) placement; and renal and respiratory failure.

### Statistical analysis

We used survey analyses for stratifying and clustering encounters for all continuous and categorical variables. The Statistical Package for the Social Sciences software program (IBM Corporation, Armonk, NY, USA) was used to perform statistical analyses. We used the chi-squared test or analysis of variance approach to identify differences in categorical variables and the two-sample t-test for the analysis of continuous variables, respectively. Logistic regression modeling was used to calculate the odds ratio (OR) for outcomes between the two study groups. This was followed by multivariate analyses to account for any confounders in the form of comorbidities between the two groups. A p-value of less than 0.05 was considered to be statistically significant. We audited the analyses using the checklist provided by the NIS to assess and confirm the data analyses were done according to the rules recommended by the NIS (https://www.hcup-us.ahrq.gov/db/nation/nis/nischecklist.jsp).

## Results

We identified a total of 7,135,090 inpatient hospitalizations in the year 2016. Among these, we further identified patients (n = 2,997) with a diagnosis of amyloidosis using the ICD-10 codes E850, E851, E852, E853, E854, E8581, E8582, E8589, and E859. We also identified patients (n = 984) with concomitant AF using the ICD-10 codes I48.0, I48.1, I48.2, I48.3, I48.4, I48.91, and I48.92. The ICD-10 codes for secondary outcomes are presented in **Supplementary Table 1**. Thus, our final sample had two study groups: patients with amyloidosis without AF (n = 2,013) and patients with amyloidosis and AF (n = 984). **Table 1** presents the background characteristics by study group. Patients with amyloidosis and AF were significantly older, with a mean age of 76.3 ± 9.7 years (p < 0.001), and included a greater proportion of men (66.2% vs. 52.8%; p < 0.001).

**Figure 1:**
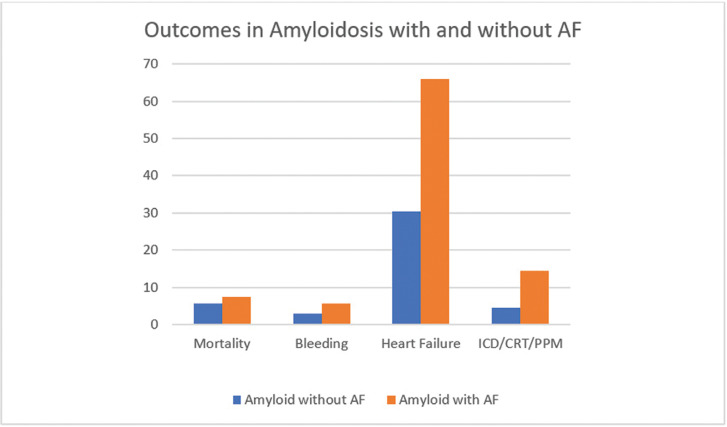
Mortality, bleeding complications, heart failure, and need for ICD/CRT/PPM implant in patients with amyloidosis with and without AF. AF: atrial fibrillation; CRT: cardiac resynchronization therapy; ICD: implantable cardioverter-defibrillator; PPM: permanent pacemaker.

**Figure 2:**
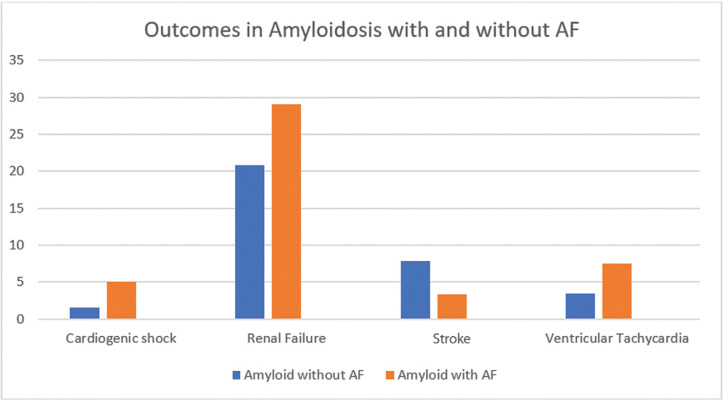
Cardiogenic shock, renal failure, stroke, and ventricular tachycardia in patients with amyloidosis with and without AF. AF: atrial fibrillation.

**Table 2** summarizes the results of logistic regression analyses used for adjusted OR calculations to control for variables in **Table 1**. Patients with amyloidosis and AF had a higher inpatient mortality rate [adjusted OR: 1.39, 95% confidence interval (CI): 1.01–1.91]. Patients with amyloidosis and AF had a greater odds of heart failure (adjusted OR: 4.61, 95% CI: 3.88–5.47), bleeding complications (adjusted OR: 2.31, 95% CI: 1.55–3.42), supraventricular tachycardia (adjusted OR: 2.02, 95% CI: 1.31–3.12), ventricular tachycardia (adjusted OR: 2.40, 95% CI: 1.68–3.41), heart block (adjusted OR: 2.36, 95% CI: 1.61–3.46), restrictive cardiomyopathy (adjusted OR: 2.66, 95% CI: 1.77–4.00), renal failure (adjusted OR: 1.59, 95% CI: 1.33–1.91), cardiogenic shock (adjusted OR: 3.77, 95% CI: 2.34–6.09), and ICD/CRT/PPM placement (adjusted OR: 3.30, 95% CI: 2.47–4.40). After controlling for confounding variables, disease severity was significantly higher in the amyloidosis with AF population, with the exceptions of stroke (adjusted OR: 0.34, 95% CI: 0.23–0.50), respiratory failure (adjusted OR: 1.08, 95% CI: 0.82–1.41), and cardiac arrest (adjusted OR: 1.57, 95% CI: 0.80–3.07). **Figures 1 and 2** present comparisons of outcomes of amyloidosis in terms of the percentage of population subset in two series.

## Discussion

The principal findings of this study were as follows: (1) there was a greater associated inpatient mortality rate in patients with amyloidosis and coexisting AF; (2) there was a higher associated cardiovascular morbidity burden in patients with amyloidosis and AF; (3) patients with amyloidosis and AF had a significantly increased odds of bleeding, but the prevalence of ischemic stroke was significantly higher in patients with amyloidosis without AF; (4) coexisting AF was associated with renal failure in patients who had compromised renal function at baseline; (5) patients with amyloidosis and AF were found to have a higher prevalence of heart block and ICD/CRT/PPM placement when compared to patients without AF; and (6) age and male sex are important and significant predictors of outcomes in patients with amyloidosis and AF.

Morbidity and mortality rates in systemic amyloidosis depend upon the extent and type of amyloid fibril deposition in the organ systems and vary from milder forms of disease to very severe forms of nephrotic syndrome, infiltrative cardiomyopathies, autonomic neuropathies leading to hypotension and diarrhea, soft tissue infiltration resulting in carpal tunnel syndrome, macroglossia, bleeding (gastrointestinal), malnutrition, and pulmonary involvement.^25–27^ AF, in general, is known to lead to a fourfold increased risk of mortality when compared to the general population after adjusting for cardiovascular comorbidities.^28,29^ AF is known to increase the risk of thromboembolism and stroke, the risk of congestive heart failure and pulmonary/renal complications, and the health care costs and to affect the quality of life.^30–34^ We found a higher inpatient mortality with an adjusted OR of 1.39 in patients with amyloidosis and coexisting AF. This observation is thought to be due to the cumulative effects on adverse outcomes like heart failure, heart block, renal failure, respiratory failure, and bleeding complications. The overall length of stay was not statistically different between the groups.

Age is considered an independent risk factor for amyloidosis and AF.^16^ Amyloid deposits in the atria with increasing age are considered a triggering factor for atrial arrhythmias and AF.^12^ These trends are consistent with our study results, as age contributed to an increased prevalence of AF in patients with amyloidosis. In previous studies, female sex was found to be protective against myocardial involvement in transthyretin-related amyloidosis.^35^ This is consistent with our study results, with an increased propensity for male sex among patients with cardiovascular involvement.

Our analyses revealed statistically significant increased prevalence rates of heart failure (66% vs. 30%; adjusted OR: 4.61, 95% CI: 3.88–5.47), restrictive cardiomyopathy (5.6% vs. 2.6%; adjusted OR: 2.66, 95% CI: 1.77–4.00), supraventricular tachycardia (4.4% vs. 2.5%; adjusted OR: 2.02, 95% CI: 1.31–3.12), ventricular tachycardia (7.5% vs. 3.5%; adjusted OR: 2.40, 95% CI: 1.68–3.41), heart block (6.8% vs. 2.8%; adjusted OR: 2.36, 95% CI: 1.61–3.46), requirement for ICD/CRT/PPM placement (14.5% vs. 4.5%; adjusted OR: 3.30, 95% CI: 2.47–4.40), and cardiogenic shock (5% vs. 1.6%; adjusted OR: 3.77, 95% CI: 2.34–6.09) in patients with amyloidosis and coexisting AF.

AF is the most common arrhythmia seen in patients with amyloidosis. An increased prevalence of ischemic stroke was recorded in patients with amyloidosis without AF; however, the association of bleeding complications was higher in patients with coexisting AF. Cardiac thrombi have been reported in cardiac amyloidosis even before the development of AF, which could be because of changes in both systolic and diastolic dynamics due to amyloid deposits.^36^ These deposits can embolize, resulting in stroke as an initial manifestation of the disease.^37^ Anticoagulation can be challenging in these patients and patients with systemic amyloidosis as they are at an increased risk of bleeding (eg, gastrointestinal hemorrhage, factor X deficiency).^38^ Patients with amyloidosis without concurrent AF had a higher prevalence of stroke (combined ischemic and hemorrhagic shock) relative to patients with coexisting AF.

Patients with amyloidosis without concurrent AF had a greater prevalence of stroke (combined ischemic and hemorrhagic shock) as compared with patients with coexisting AF. Amyloidosis increases the risk of intra-atrial and intracardiac thrombi, even without coexisting AF.^36,37^ This could possibly explain the increased prevalence of stroke in patients without concurrent AF. Moreover, patients with amyloidosis and coexisting AF, irrespective of their CHADS_2_-VASc score, are supposed to be on anticoagulation; this could very well explain the reduced risk of stroke in this group, who are likely receiving anticoagulation, in our study. Patients with amyloidosis are at an increased risk of bleeding, including mainly gastrointestinal bleeding, and factor X deficiency.^39^ Moreover, patients with coexisting AF were also found to exhibit an increased risk of bleeding as they were on anticoagulation for thromboembolic prevention. This could possibly explain the increased risk of bleeding in amyloidosis patients with coexisting AF.

Cardiac involvement in amyloidosis can cause infiltration of the conduction system and result in heart block requiring ICD/CRT/PPM placement as evident from our results; however, concurrent refractory AF can worsen the prevalence of ICD/CRT/PPM implantation due to procedures such as AV nodal ablation, making patients PPM-dependent and resulting in cardiomyopathies, heart failure, and increased mortality rates.^8,9,40–42^

Renal amyloid deposition manifests as albuminuria and progresses to nephrotic-range proteinuria, which is almost always diagnosed in advanced forms of the disease. Amyloidosis manifesting clinically with renal involvement is considered very rare.^26,43–45^ AF with a loss of atrial kick, which contributes to 20% to 25% of the cardiac output, can lead to a fall in blood pressure and decreased perfusion to the kidneys, further worsening the renal dysfunction.^45^ Our analyses showed that coexisting AF is associated with worse renal outcomes in patients with amyloidosis (29% vs. 20.8%) as compared with in those without coexisting AF.

### Limitations

The inherent nature of a cross-sectional study did not allow us to calculate incidence and rate ratios. Reliance on the HCUP database can also have limitations of its own, eg, due to the select group of patients included in the database. It could not be determined whether the patients had paroxysmal versus persistent AF. Moreover, anticoagulation status in both the groups could not be determined either. Another limitation to the study was that there was not a single case with a primary diagnosis of transthyretin cardiomyopathy during the years 2016 and 2017 and this resulted in the limitation of not being able to conduct a subgroup analysis of patients with transthyretin cardiomyopathy. Additionally, we took a composite of ischemic and hemorrhagic stroke, but it would have been ideal to have considered them as separate entities. Moreover, the NIS has inherent limitations of a lack of distinction between new and chronic diagnoses and between comorbidities and complications.^22,23^ Still, the increased mortality rates and significant trend toward other outcomes in the AF and amyloidosis groups can serve as a platform for further research. Given the underdiagnosed nature of amyloidosis, new ideas and management strategies should be formulated to enhance future practice.

## Conclusion

Amyloidosis with AF is associated with a higher inpatient mortality rate and other adverse clinical outcomes. Optimizing the management of AF in patients with amyloidosis may help to improve outcomes. Further studies in the future can help us to understand the underlying pathogenesis of these outcomes and also help devise strategies to improve outcomes.

## Figures and Tables

**Table 1:** Background Characteristics by Study Group

	Amyloidosis Alone (n = 2,013)	Amyloidosis with AF (n = 984)	p-value
Age (years)	71.3 ± 12.1	76.3 ± 9.7	< 0.001
Sex (% male)	52.8%	66.2%	< 0.001
Race (% non-white)	35.5%	33.3%	0.469
County location* (% completely rural)	5.1%	4.5%	0.268
County income (% lowest quartile)	27.5%	36.3%	0.036
Hospital bed size** (% small)	11.1%	13.0%	0.322

AF: atrial fibrillation.

*Based on the National Center for Health Statistics Urban–Rural Code; % non-metropolitan, non-micropolitan.

**Definitions of hospital bed size categories vary by geographic region of the country, rural or urban setting, and whether the facility is a teaching hospital.

**Table 2:** Outcomes and Comorbidities of Amyloidosis with and Without AF

	Amyloidosis Alone (n = 2,013)	Amyloidosis with AF (n = 984)	OR (95% CI)	aOR* (95% CI)
Stroke	7.9%	3.4%	0.39 (0.27–0.58)	0.34 (0.23–0.50)
Heart failure	30.4%	66.0%	4.47 (3.80–5.27)	4.61 (3.88–5.47)
Bleeding	2.8%	5.7%	2.10 (1.44–3.07)	2.31 (1.55–3.42)
Ventricular tachycardia	3.5%	7.5%	2.28 (1.63–3.20)	2.40 (1.68–3.41)
Heart block	2.8%	6.8%	2.60 (1.80–3.75)	2.36 (1.61–3.46)
Cardiac arrest	1.1%	1.6%	1.42 (0.75–2.71)	1.57 (0.80–3.07)
Restrictive cardiomyopathy	2.6%	5.6%	2.22 (1.51–3.27)	2.66 (1.77–4.00)
Cardiogenic shock	1.6%	5.0%	3.16 (2.01–4.98)	3.77 (2.34–6.09)
ICD/CRT/PPM placement	4.5%	14.5%	3.59 (2.71–4.74)	3.30 (2.47–4.40)
Renal failure	20.8%	29.1%	1.59 (1.33–1.90)	1.59 (1.33–1.91)
Respiratory failure	9.5%	9.8%	1.03 (0.80–1.34)	1.08 (0.82–1.41)
Inpatient mortality	5.6%	7.4%	1.34 (0.99–1.83)	1.39 (1.01–1.91)
Length of stay (includes only those patients who survived to discharge)	7.4 ± 9.2 days	7.8 ± 12.9 days	—**	—

AF: atrial fibrillation; aOR: adjusted odds ratio; CI: confidence interval; CRT: cardiac resynchronization therapy; ICD: implantable cardioverter-defibrillator; OR: odds ratio; PPM: permanent pacemaker.

*Adjusted for age, sex, and county income.

**Continuous variable; t-test nonsignificant (p = 0.301).

**Supplementary Table 1:** ICD-10 Codes Used

Diagnosis	ICD-10 Codes
Atrial fibrillation	I48.0, I48.1, I48.2, I48.3, I48.4, I48.91, and I48.92
Amyloidosis	E854, E850, E851, E852, E853, E854, E8581, E8582, E8589, and E859
Heart block	I445, I452, I4430, I441, I447, I455, I444, I442, I440, I4439, I4460, I4469, I450, I4510, I4519, I453, and I454
Cardiogenic shock	R57.0
Cardiac arrest	I46, I468, I469, and I462
Stroke	All I63 codes excluding I6389 and I639
CIED indication	0JH604Z, 0JH634Z, 0JH804Z, 0JH834Z, 0JH605Z, 0JH635Z, 0JH805Z, 0JH835Z 0JH606Z, 0JH636Z, 0JH806Z, 0JH836Z 0JH60PZ, 0JH63PZ, 0JH80PZ, 0JH83PZ 0JH608Z, 0JH638Z, 0JH808Z, and 0JH838Z
Bleeding complications	K29.01, K62.5, K31.811, K57, K29, K25, K26, K27, K28, I85.01, and N93
Congestive heart failure	I50
Restrictive cardiomyopathy	I42.5
Renal failure	N17–N19
Respiratory failure	J96.92
Ventricular tachycardia	I47.2

CIED: cardiac implantable electronic device.
